# Dementia Advocacy in Action: Examining Social Media During World Alzheimer’s Month

**DOI:** 10.1093/geroni/igaf122.2574

**Published:** 2025-12-31

**Authors:** Juanita-Dawne Bacsu, Raymond J Spiteri, Sarah Fraser, Allison Cammer, Alison L Chasteen, Zahra Rahemi, Kate Nanson, Ali Akbar Jamali

**Affiliations:** Thompson Rivers University, Kamloops, British Columbia, Canada; University of Saskatchewan, Saskatoon, Saskatchewan, Canada; University of Ottawa, Ottawa, Ontario, Canada; University of Saskatchewan, Saskatoon, Saskatchewan, Canada; University of Toronto, Toronto, Ontario, Canada; Clemson University, Clemson, South Carolina, United States; Thompson Rivers University, Kamloops, British Columbia, Canada; University of Saskatchewan, Saskatoon, Saskatchewan, Canada

## Abstract

Dementia awareness campaigns play a vital role in reducing stigma and improving the quality of life of people with dementia. However, there is a paucity of research on advocacy strategies to support dementia awareness, especially on social media. This presentation aims to: i) identify dementia advocacy strategies used to increase awareness on social media during World Alzheimer’s Month; and ii) understand the role of social media in dementia awareness campaigns. Using data from the social media platform X, posts were scraped from September 1 to September 30, 2022. Filters were used to screen for duplicate posts, non-English content, and reply posts with missing content. The remaining 1,981 relevant posts were examined using thematic analysis to identify prominent dementia advocacy strategies. During our data analysis, steps were taken to support rigor such as using a standardized codebook, practice exercises, intercoder reliability checks, and team discussions to oversee coding disagreements. Based on our analysis, we identified four main advocacy strategies: i) myth-busting to address false information; ii) political advocacy to support dementia care reform; iii) tailoring messages to ethnic and cultural groups; and iv) amplifying personal narratives and lived experience of dementia. Although a range of strategies were identified, further research is needed to evaluate the effectiveness of awareness campaigns in addressing dementia-related stigma. The findings from our study have implications for community leaders, health professionals, and policymakers who are working to increase dementia awareness. Our findings provide vital insight to enhance national-level dementia advocacy and awareness campaigns on social media.

